# Herpes Zoster Before the First Birthday: A Case Report and Primary Care Perspective

**DOI:** 10.7759/cureus.88910

**Published:** 2025-07-28

**Authors:** Rui Guilherme Costa, Mariana Bernardo

**Affiliations:** 1 USF Manuel Cunha, Family Health Unit, Coimbra Local Health Unit, Coimbra, PRT; 2 USF Terras de Cantanhede, Family Health Unit, Coimbra Local Health Unit, Coimbra, PRT

**Keywords:** herpes zoster virus, pediatric dermatology, pediatric infectious disease, shingles, varicella-zoster

## Abstract

This case illustrates a rare presentation of herpes zoster (HZ) in infancy. A six-month-old male infant was observed during a routine health surveillance visit with a four-day history of vesicular rash localized to the right upper limb and dorsum, following a dermatomal distribution (C7-C8). The child had previously had varicella at 48 days of age. He was afebrile, asymptomatic, and in good general condition. Clinical findings and medical history supported a diagnosis of HZ. Given the mild presentation and the time elapsed since onset, a conservative management approach with clinical monitoring was adopted. Complete resolution occurred within 10 days. This case provides an example of HZ in early infancy, identified during routine follow-up, and managed successfully without complications. This case reinforces the importance of clinical suspicion in atypical age groups and highlights the central role of the family physician in longitudinal care and early recognition of uncommon pediatric conditions.

## Introduction

Herpes zoster (HZ) is a viral infection caused by the reactivation of the varicella-zoster virus (VZV), a neurotropic herpesvirus that remains latent in the sensory or cranial nerve ganglia following primary infection, typically manifesting as varicella (chickenpox) [1,2]. While the primary infection is highly contagious and occurs predominantly during childhood, viral reactivation is more frequent after the age of 45, particularly in elderly or immunocompromised individuals (due to malignancy, HIV infection, or immunosuppressive therapy) [3].

Unlike primary varicella infection, which spreads through airborne transmission and has high contagiousness, HZ is only moderately contagious. Transmission occurs through direct contact with vesicular fluid, and airborne or indirect transmission is rare. Susceptible individuals infected in this context develop varicella, not HZ [4].

HZ is typically regarded as a disease of adulthood, with a marked increase in incidence after the sixth decade of life. Among individuals aged over 75 years, the incidence reaches 4.2-4.5 per 1,000 person-years, whereas, in children aged 0-14 years, it is approximately 0.45 per 1,000 person-years. In children under five years of age, the incidence is estimated at around 20 per 100,000 person-years, likely underestimated due to mild and self-limiting clinical presentations [5].

Clinically, HZ evolves through three distinct stages. The prodromal stage (one to five days) is characterized by localized pain, burning, or altered sensitivity (hypo- or hyperalgesia) in the affected dermatome. This is followed by the acute eruptive stage, marked by a unilateral vesicular rash, typically involving one to three contiguous dermatomes corresponding to the infected sensory root [1]. Systemic symptoms such as low-grade fever, malaise, or headache may accompany the eruption. Between 7 and 10 days later, the resolution stage begins, with the formation of crusts and progressive healing of the lesions.

Complications may arise during or after the clinical course, depending on the dermatome affected and the host’s immune status. These include HZ ophthalmicus, Bell’s palsy, encephalitis, disseminated zoster in immunocompromised patients, and secondary bacterial infection of the skin lesions [6].

In pediatric patients, however, HZ typically follows a more benign course, with milder pain, faster healing, and a lower risk of complications [7]. The unusual occurrence of HZ in infants poses a diagnostic challenge, as the early manifestations may be nonspecific and the clinical suspicion is generally low in this age group.

The HZ vaccine is not licensed or recommended for use in pediatric patients. In this age group, prevention focuses primarily on varicella vaccination, which targets the primary VZV infection. In Portugal, varicella vaccination is not included in the National Vaccination Program (PNV) but is available by medical prescription and is indicated in selected circumstances [8].

By significantly reducing the incidence of primary VZV infection, varicella vaccination indirectly decreases the future risk of VZV reactivation and subsequent HZ - even in pediatric populations.

According to the joint recommendation of the Portuguese Society of Pediatric Infectious Diseases and the Portuguese Society of Pediatrics, varicella vaccination is advised from age 11-13 in the following cases: non-immune individuals in high-risk occupations (e.g., healthcare workers, teachers, childcare staff); non-immune women planning pregnancy; parents of young, unvaccinated children; and individuals who are in regular contact with immunosuppressed patients. Varicella vaccination also plays a role in post-exposure prophylaxis, especially in moderate to severe cases, if administered within 72 hours of exposure [8].

As per these same guidelines, the varicella vaccine is contraindicated in children under nine months of age, immunocompromised individuals, pregnant women, persons with a history of allergy to vaccine components, patients with moderate-to-severe acute illness, and those undergoing treatment with salicylates [8].

This case report aims to describe the presentation of HZ in a six-month-old infant, outlining the clinical findings and discussing potential etiopathogenic mechanisms, as well as the diagnostic and therapeutic approach appropriate for this age group.

## Case presentation

A six-month-old male infant, born to Brazilian parents via cesarean section after a pregnancy complicated by insulin-treated gestational diabetes, was evaluated during a routine well-child visit. His medical history included two hospital admissions during the neonatal period: the first at 42 days of life for pertussis, treated with a seven-day course of clarithromycin; and the second at 48 days of life for varicella, following household exposure from an infected four-year-old sibling, for which he received intravenous acyclovir (20 mg/kg/dose every six hours).

As the pregnancy was not monitored in Portugal - since the family remained in Brazil until the later stages of gestation - no documentation was available regarding the mother’s immunity to varicella, either through prior infection or vaccination. It was also not possible to confirm whether she received Tdap (tetanus, diphtheria, and pertussis) vaccination during pregnancy.

At the time of the visit, the child was up to date with the national immunization schedule, which does not include the varicella vaccine. Growth and neurodevelopment were appropriate for age. He had been exclusively breastfed during the first six months of life, after which complementary feeding was introduced while breastfeeding continued. The only ongoing medication was daily colecalciferol supplementation.

During the anamnesis, the mother reported the appearance of skin lesions on the right upper limb and right dorsal region, first noted four days prior. Due to the child’s age, assessment of prodromal symptoms was limited; however, the mother denied any irritability or signs of discomfort. There was no associated fever, changes in general condition, appetite, or bowel or urinary habits.

**Figure 1:**
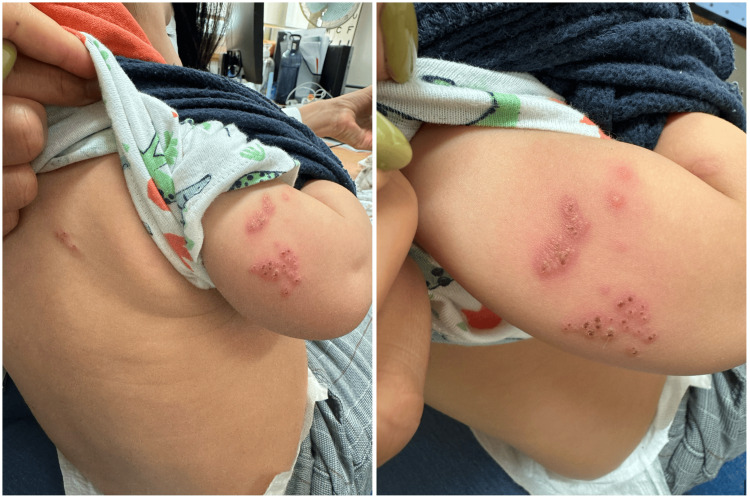
Zoster lesions in the posterior region of the right upper limb and ipsilateral dorsal region.

On physical examination, grouped and confluent vesiculobullous lesions with serous content and an erythematous base were observed on the posterior aspect of the right upper limb, extending over approximately 10 cm. A single vesicle with similar features was also noted on the right dorsal region (Figure 1). These lesions corresponded anatomically to dermatomes C7-C8. The infant was in good general condition, afebrile, and without other relevant findings. No ophthalmological, otological, or neurological abnormalities were identified on examination.

Given the clinical presentation, dermatomal distribution, and prior history of varicella infection, the pediatric team at the referral hospital was contacted. Based on clinical images and history, a diagnosis of HZ was made.

Considering the absence of systemic signs, the child’s good general status, and the fact that the lesions had appeared more than 72 hours earlier, a conservative approach was adopted, consisting of close clinical follow-up and supportive care only.

**Figure 2:**
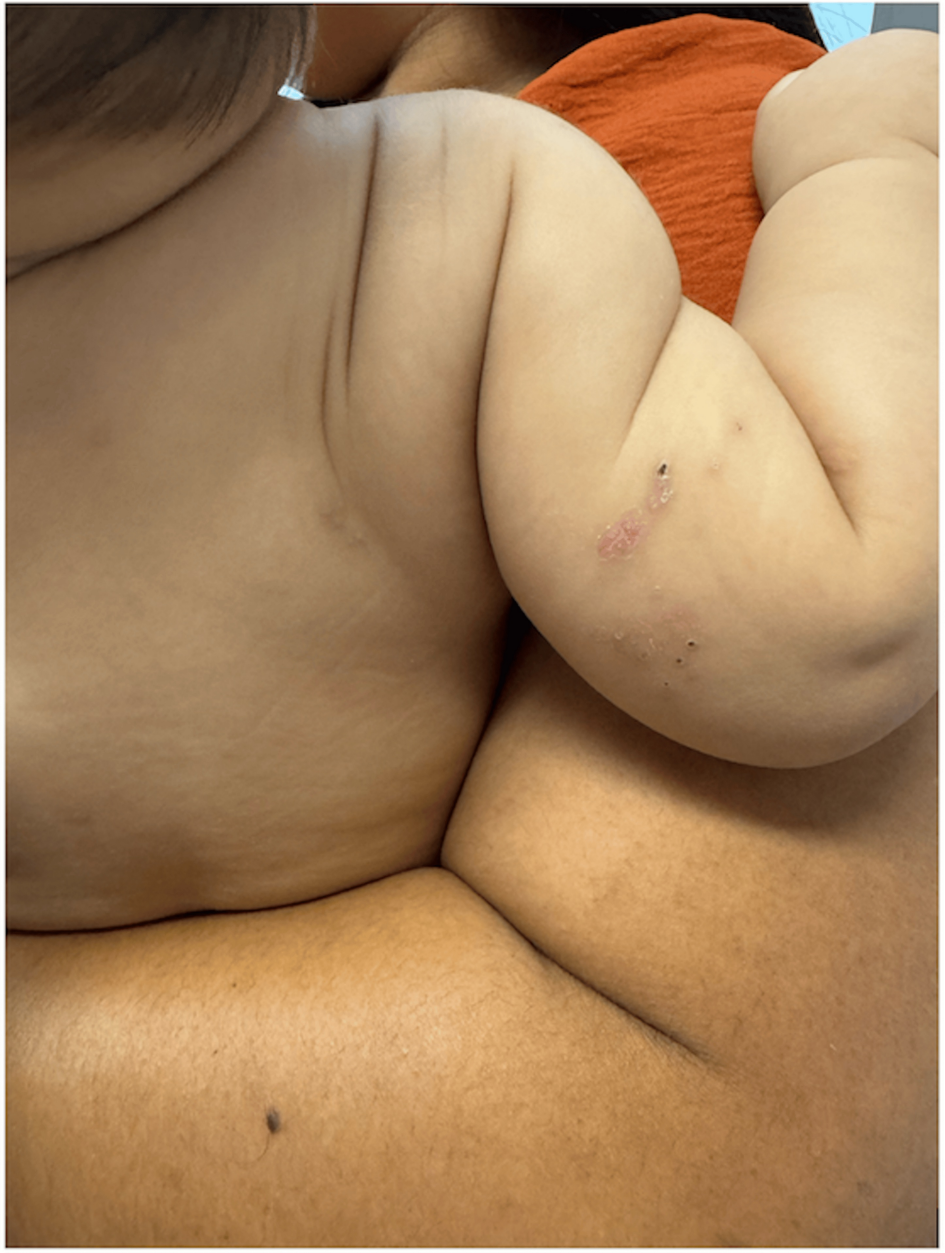
Healed zoster lesions observed at reassessment consultation 10 days later.

At the 10-day follow-up visit, near-complete resolution of the cutaneous lesions was observed, with only a residual crust on the right upper limb (Figure 2). The infant remained asymptomatic and clinically stable, with no signs of complications.

## Discussion

Several pediatric studies identify varicella infection before one year of age as the main risk factor for HZ in early childhood [2]. Among infants with HZ before 12 months, two-thirds are associated with maternal varicella during pregnancy (intrauterine infection), while one-third of the cases result from postnatal infection [3]. In this case, the child had documented postnatal varicella at one month of age due to sibling exposure.

Another relevant variable is the maternal immunization status, since breastfeeding may allow for the transfer of protective maternal antibodies. These antibodies may either prevent infection or attenuate its severity in case of transmission. In this case, both the mother’s vaccination status and her history of varicella were unknown. Given the potential risks of VZV infection during pregnancy, it is important to assess VZV seroprotection in women of childbearing age planning to conceive, as vaccination can be offered preconceptionally.

Considering the high contagiousness of VZV, it is crucial to emphasize to family members the need to protect more vulnerable individuals (newborns, immunocompromised persons, pregnant women, etc.) when a household member, as in this case, the older sibling, is infected with varicella.

The diagnosis of HZ was considered the most likely. Diagnosis of HZ is typically clinical, based on the appearance of vesicular rash with an erythematous base, initially presenting as papules that evolve into vesicles, pustules, and crusts over 7-10 days [9]. Complete resolution usually occurs within one to three weeks [1]. The rash may be preceded by pain, erythema, fever, irritability, local hypersensitivity, or lymphadenopathy [1,9]. HZ frequently involves cranial, cervical, and thoracic dermatomes [2,3].

The VZV establishes latency in the dorsal root ganglia after primary infection, typically at the level corresponding to the initially affected dermatome. Its subsequent reactivation, usually triggered by immunosuppression, leads to HZ manifestations restricted to that same dermatome. Thus, a history of varicella and a dermatomal distribution should raise diagnostic suspicion.

The absence of systemic signs (e.g., fever, prostration, or irritability), the favorable clinical course, and the overall normal physical examination supported the diagnosis of a localized and self-limited form of HZ.

Pediatric cases of HZ often present with milder, nonspecific symptoms, making misdiagnosis as impetigo or other common dermatoses more likely, which requires the exclusion of other relevant differential diagnoses [3,5]. Bullous impetigo, more common in pediatric age, may present with vesicles and honey-colored crusts but usually evolves more rapidly and lacks a localized dermatomal pattern. Cutaneous herpes simplex, although typically affecting orofacial or genital areas, may occasionally appear elsewhere, but it rarely follows a dermatomal distribution. Other possible differential diagnoses include hypersensitivity reactions, such as fixed drug eruptions, and insect bites, though their morphology, topography, and the absence of pruritus are less compatible. Rare entities such as dermatitis herpetiformis, usually associated with celiac disease and characterized by symmetric, pruritic lesions, are highly unlikely in this age group [10].

When needed, viral culture or PCR for VZV can confirm the diagnosis [3]. In immunocompetent children, early postnatal or intrauterine exposure to VZV may lead to HZ due to immaturity of the immune system [3]. Unlike in adults, pediatric HZ does not typically suggest underlying immunodeficiency. Immunologic workup is unnecessary in previously healthy children unless the presentation is atypical, severe, or recurrent, especially if no prior VZV infection is documented [9]. In such cases, the first step is to rule out acquired immunodeficiencies (e.g., HIV) or malignancies (e.g., leukemia and Hodgkin’s lymphoma), followed by basic immunologic screening and further evaluation depending on findings.

In immunocompetent children, HZ usually follows a benign and self-limiting course, and systemic antiviral therapy is generally unnecessary when diagnosis occurs more than 72 hours after the onset of lesions [11]. Although some complications may arise later, current evidence does not support a clear benefit from late initiation of antiviral therapy in preventing them, and clinical surveillance remains the most widely accepted approach [12]. In this case, the mild presentation, preserved general condition, and delayed presentation justified a watchful waiting approach, agreed upon by parents, the family physician, and the consulting pediatrician.

Antiviral treatment of HZ, oral acyclovir (up to 80 mg/kg/day in four to five divided doses), in immunocompetent children should be limited to treating ophthalmic zoster and zoster that at onset causes a moderate to severe rash and pain [1,2,3,9]. Severe cases may require intravenous administration [1,9]. In these contexts, systemic corticosteroids may be considered as an adjuvant therapy, particularly in adolescents and adults, to reduce inflammation and improve neurological recovery. However, evidence supporting their use in pediatric age is limited, and their indication should be evaluated on a case-by-case basis [10]. Topical acyclovir, although used for other uncomplicated herpes infections, has limited efficacy in HZ, including in pediatric patients, and is not recommended as a standalone treatment [11].

Supportive care in pediatric HZ includes symptomatic relief with analgesics and topical care to reduce discomfort and prevent bacterial superinfection. Emollients should be simple and free of disinfectants, as these may cause additional irritation. Basic hygiene measures, such as regular nail trimming and gentle skin cleansing, are also recommended to promote healing and reduce the risk of transmission.

Post-herpetic neuralgia is rare in children [2]. Its diagnosis in young children is challenging due to the subjective nature of the symptoms and limited verbal expression. When confirmed, treatment should be tailored to age and symptom severity, beginning with first-line analgesics such as paracetamol or ibuprofen. In this case, no complications, including post-herpetic neuralgia, were observed.

Clinical follow-up in pediatric HZ aims to monitor the resolution of skin lesions, prevent secondary infections, and ensure early identification of neurological or ophthalmologic complications, which, though rare, are the most significant in this age group. In otherwise healthy children with localized forms, follow-up is generally limited to periodic clinical reassessment. When managed in primary care, these children are also covered by the routine Portuguese National Child Health Surveillance Program, allowing for continued active monitoring. Warning signs such as facial asymmetry, gait disturbance, prostration, ocular pain, photophobia, or oculomotor abnormalities should prompt urgent reassessment and, if needed, referral to specialized care.

## Conclusions

This case illustrates that HZ can occur even in early infancy, particularly following recent postnatal varicella infection. The benign clinical course, absence of systemic symptoms, and favorable outcome supported the safety of a conservative, watchful waiting approach in this immunocompetent child. The diagnosis was made during a routine health surveillance visit, reinforcing the importance of continuity of care and the clinician’s familiarity with the patient’s medical history.

Beyond clinical management, this case also highlights key prevention strategies. Varicella vaccination - although not yet universally included in all national immunization programs - remains the primary measure to prevent both primary infection and future reactivation of the VZV. In households with more than one child, consideration should be given to vaccinating unexposed or unvaccinated siblings, as well as assessing maternal serostatus in women of childbearing age who are planning future pregnancies. Families should also be educated on hygienic measures and infection control practices to reduce the risk of transmission to vulnerable contacts, such as newborns, pregnant women, or immunocompromised individuals. Ultimately, this case underscores the relevance of early identification, longitudinal patient monitoring, and preventive health strategies within pediatric primary care - especially when facing uncommon but clinically significant viral reactivations in early life.
